# Can diabetic peripheral neuropathy influence the final outcome of physical performance after a combined training program in older adults? A double‐blind quasi‐experimental study

**DOI:** 10.14814/phy2.70755

**Published:** 2026-06-04

**Authors:** Matheus Henrique dos Santos Lino, Marcus Vinicius Grecco, Eneida Yuri Suda, Sara Rodrigues Rosado, André Luiz de Seixas Soares, Adriana Machado Saldiba de Lima, Guilherme Carlos Brech, Julia Maria D'Andréa Greve, Angelica Castilho Alonso

**Affiliations:** ^1^ Graduate Program in Aging Sciences University São Judas Tadeu São Paulo Brazil; ^2^ The Movement Study Laboratory of the Hospital das Clínicas The School of Medicine of the University of São Paulo São Paulo Brazil; ^3^ Graduate Program in Physical Therapy University Ibirapuera São Paulo Brazil; ^4^ The Undergraduate Nursing Program University Center São Camilo São Paulo Brazil; ^5^ Georgia Prevention Institute, Medical College of Georgia Augusta University Augusta Georgia USA

**Keywords:** aged, diabetic neuropathies, exercise, type 2 diabetes mellitus

## Abstract

This study investigated the influence of diabetic peripheral neuropathy (DPN) on physical performance in older adults with type 2 diabetes mellitus (T2DM) following a combined training (CT) program. A total of 51 older adults participated in the study divided into DPN (*n* = 24) and non‐DPN (*n* = 27) groups. Muscle strength was assessed by handgrip strength; physical performance by the Short Physical Performance Battery, and the Senior Fitness Test (SFT); and balance by the Timed Up and Go (TUG) test, with and without a cognitive task. The CT program lasted 12 weeks, with two weekly sessions including resistance exercises for major muscle groups and high‐intensity interval aerobic training on a stationary bicycle. Compared with the DPN group, the non‐DPN group showed greater improvements in agility and dynamic balance in the SFT (*p* = 0.010) and TUG (*p* = 0.018). BMI and diabetes duration explained 15% of the variance in the 30‐s chair stand. DPN presence explained 13% of the variance in SFT agility and balance and 20% in TUG, while age and BMI explained 12% of the variance in the 4 m walk test. DPN attenuates the response to combined training, particularly affecting functional outcomes related to mobility and balance.

## Graphical abstract

Illustration of the main findings of the study.

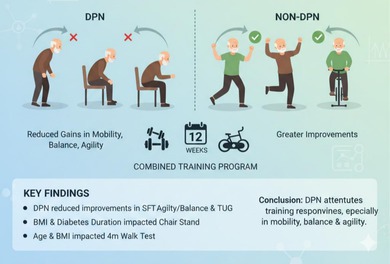

## 1 INTRODUCTION

Diabetic peripheral neuropathy (DPN) is one of the most prevalent complications of type 2 diabetes mellitus (T2DM), characterized by symptoms such as an increased risk of severe injuries, loss or reduction of sensation, tingling in the extremities, heightened instability and imbalance, and impairments in physical performance. Contributing factors to the pathogenesis of DPN include hyperglycemia, nitrosative and oxidative stress, mitochondrial dysfunction, activation of the polyol pathway, accumulation of advanced glycation end products (AGEs), and increased neural irritability, resulting in degeneration of neurons and axons in sensory and motor nerves (Abuhay et al., 2022; Kluding et al., 2017; McMorrow et al., 2022).

The DPN follows a specific pattern: initially, the sensory nerves are affected, followed by the motor nerves, in a process known as “dying‐back neuropathy” in which the distal axons degenerate before the complete destruction of the nerve cell body. This pattern results from a disturbance in the production of proteins and substances essential for axons, being more common in longer axons (Seyedizadeh et al., 2020).

DPN is associated with neurogenic muscle atrophy, leading to reductions in strength, speed, and muscular endurance, particularly in the lower limbs. These impairments stem from the degeneration of motor and sensory nerve fibers, leading to neural fatigue, proprioceptive deficits, and postural instability. The loss of lower limb muscle strength is considered one of the main factors contributing to imbalance, gait alterations, and increased risk of falls in individuals with T2DM (Khan et al., 2022; Seyedizadeh et al., 2020).

Given these impairments, physical performance becomes a key target for intervention. Multiple lines of evidence suggest that structured exercise programs, especially those combining resistance and aerobic training, provide significant benefits for the overall physical performance of this population. These improvements include enhanced muscle strength, functional capacity, and balance, with the potential to reduce fall risk and promote independency (Ghanavati et al., 2012; Venkataraman et al., 2019).

Until 2009, the American Diabetes Association (American College of Sports Medicine, 2009) imposed several restrictions on physical exercise participation for individuals with DPN, due to the lack of information regarding the most appropriate type of activity for those at greater risk of developing foot injuries, peripheral arterial disease, or foot deformities. This was attributed to the reduced or absent protective sensation, leading to insensitivity to trauma and making these individuals particularly susceptible to complications. However, over the past decade, numerous studies have highlighted the benefits of exercise for individuals with DPN. These studies have demonstrated positive adaptations in response to load‐bearing exercises, including peripheral neuroplasticity, which have since been incorporated into the guidelines of several medical societies specializing in diabetes (Kluding et al., 2017; Streckmann et al., 2022; Van Netten et al., 2024).

According to the ADA 2023 Standards of Care (ElSayed et al., 2023), it is crucial to understand the types of exercises that can provide benefits without worsening conditions such as DPN. Light to moderate activities, such as walking, cycling, or swimming, have been shown to be beneficial for people with DPN. Emphasis is recommended on light to moderate aerobic activities together with resistance training (RT), with a gradual increase in exercise duration and load.

The study conducted by Seyedizadeh et al. (2020) is one of the few that evaluated the effects of combined training (CT) (resistance‐aerobic) on kinase levels and physical function in patients with DPN. Although the results did not show a significant decrease in kinase levels and aerobic endurance after 8 weeks of training, a significant increase in lower limb strength was observed. These findings suggest that even small changes resulting from CT can be beneficial, especially considering the progressive nature of DPN. However, further research in this field is needed to better understand its effects and conduct studies with longer training durations.

DPN is a common and debilitating complication in older adults with T2DM, affecting sensation, mobility, and functional capacity. Its impact on exercise response is not well understood, yet understanding this is crucial for designing effective interventions to improve functionality, prevent complications, and enhance quality of life.

The present study aimed to evaluate the influence of DPN on the physical performance of older adults living with T2DM following participation in a CT program.

## 2 METHODS

### 2.1 Study design and ethics

This was a double‐blind, nonrandomized (quasi‐experimental) clinical trial, approved by the Ethics Committee of the Universidade São Judas Tadeu under protocol number CAAE 70431623.20000.008, opinion number 6.223.888. Moreover, it conforms to the Helsinki Declaration's standards for research involving human subjects. This clinical investigation is formally registered in the Brazilian Clinical Trials Registry (ReBEC) under the number RBR‐6d43xbb. All participants provided written informed consent before participating in any tests or interventions in the present study.

### 2.2 Study location

The study was conducted at the São Judas Tadeu University (USJT) in partnership with the Movement Study Laboratory (LEM) of the Institute of Orthopedics and Traumatology at the Hospital das Clínicas of São Paulo (IOT‐HC‐FMUSP).

### 2.3 Participants and recruitment

Initially, 64 participants were recruited for the study; after eligibility and exclusion assessments, the final sample consisted of 51 participants of both sexes, with T2DM and aged between 65 and 79 years, who were divided into two groups according to the presence or absence of DPN: a group without diabetic peripheral neuropathy (non‐DPN) and another with diabetic peripheral neuropathy (DPN).

The sample size calculation was based on a pilot study with 10 participants, using one of the primary outcomes as the main variable: the total score of the Short Physical Performance Battery (SPPB), which ranges from 0 to 12 points. A two‐tailed hypothesis was considered, with a significance level (*α*) of 5% and a type II error (*β*) corresponding to a statistical power of 80%. Based on these parameters, a minimum of 21 participants per group was estimated. To mitigate potential sample losses due to dropouts, the recruitment target was increased by 25%; recruitment was planned.

The inclusion criteria were: diagnosis of T2DM for more than 2 years, with a stable medication dose (oral antidiabetics, insulin, or a combination of both) for over 1 year; HbA1c between 6.0% and 9.0%; absence of resistance or combined training in the last 6 months, a score above 26 points on the Montreal Cognitive Assessment (MoCA) for individuals with at least 8 years of education; no other orthopedic injuries, pain, any type of disabling disease, or previous surgeries that could prevent physical training; and, in the case of hypertensive patients, controlled blood pressure. Exclusion criteria included the inability to complete the assessments and reassessments.

### 2.4 Procedures

Participants were referred for a clinical evaluation conducted by a physician to determine eligibility and order laboratory tests.

### 2.5 Assessments

#### 2.5.1 Laboratory analysis

The older adults were instructed to attend the clinical analysis laboratory after an 8–12 h fast. Peripheral blood samples (20 mL) were collected from the median basilic or cephalic vein at baseline and at the end of the intervention using tubes containing EDTA as an anticoagulant. Whole blood with EDTA was centrifuged to obtain plasma, which was stored at −20°C. The plasma samples were used to determine glucose, insulin, fructosamine, and HbA1c concentrations using commercial kits, in accordance with the manufacturer's instructions (Labtest, Minas Gerais, Brazil).

#### 2.5.2 Sociodemographic characterization

Sociodemographic data were collected through questions related to sex, age, duration since clinical diagnosis of T2DM, physical activity, Body Mass Index (BMI), among other relevant variables.

#### 2.5.3 Montreal cognitive assessment (MoCA)

The MoCA instrument was applied to assess global cognition, including visuospatial skills, executive function, language, memory, attention and orientation, calculation, and abstraction. Its score ranges from 0 to 30, with scores below 26 points among individuals with a minimum of 8 years of education, indicating the presence of cognitive impairment (Paraizo Mde et al., 2016).

#### 2.5.4 Assessment of neuropathy signs and symptoms

The Brazilian version of the Michigan Neuropathy Screening Instrument (MNSI‐Brasil) (Oliveira et al., 2016) was applied, consisting of two parts: the first is a self‐administered questionnaire completed by the patient, and a healthcare professional conducted the second.

##### Questionnaires on signs and symptoms of the lower limbs

The questionnaire consists of 15 self‐report questions addressing aspects such as pain, tingling, numbness, cramps, and changes in sensitivity in the feet and legs. Participants answered “yes” or “no” to each question based on the presence or absence of symptoms during recent months.

##### Tactile sensitivity

This test is performed by inspecting three specific points on the feet using a 10 g Semmes‐Weinstein nylon monofilament to check for the absence or presence of plantar sensitivity. With the participant comfortably lying on a stretcher in the dorsal decubitus position, the test was first demonstrated on the hand for better understanding. Then, the participant was instructed to close their eyes while the monofilament was touched three times on the plantar region, in random order, at the following points: the first toe and the first and fifth metatarsals. The participant was asked to verbally indicate when they felt the touch (International Working Group on the Diabetic Foot, 2019).

##### Vibratory sensitivity

The vibratory sensitivity test was performed bilaterally using a 128 Hz tuning fork placed on the dorsal side of the hallux, at the metatarsophalangeal joint. With the participant in the dorsal decubitus position, the test was first demonstrated on the hand before being applied to the plantar region in random order, with eyes closed. The vibration, lasting about 10 s, was rated by the participant as “felt strongly,” “felt weakly,” or “not felt,” with one attempt per region. It was considered “present” if the examiner still perceived the vibration for less than 10 s after the participant's report, “reduced” if lasting 10 s or more, and “absent” if not detected (International Working Group on the Diabetic Foot, 2019).

#### 2.5.5 Classification of diabetic peripheral neuropathy

To classify participants with diabetic DPN, a fuzzy logic‐based classification system was used. This system uses as input the presence of DPN symptoms based on the MNSI, tactile sensitivity (the number of insensitive areas on the plantar surface detected with the 10‐g Semmes‐Weinstein monofilament), and vibratory sensitivity (assessed with the 128 Hz tuning fork). The system processes each input variable using fuzzy sets and, through the Mamdani inference process, performs a combinatorial analysis of these variables, associating these combinations with fuzzy output sets. Then, the system applies the centroid defuzzification method to convert the resulting output sets into a numerical value (0–10), corresponding to the degree of membership to the set and, therefore, a score representing the degree of neuropathy (Watari et al., 2014).

The “neuropathy degree score” was used to define the cutoff point: DPN absent (<2.5) and DPN present (≥2.5). This score ranges from 0 to 10, with higher values indicating greater severity of neuropathy (Suda et al., 2016).

#### 2.5.6 Assessment of handgrip strength (HGS)

For this assessment, a Jamar® manual dynamometer was used. The participant remained seated in a chair without armrests, with feet flat on the floor and hips and knees flexed at 90°. Shoulders were abducted and in a neutral rotation position, elbows flexed at 90°, and forearms and wrists in a neutral position. Hands were alternated with each maneuver, with 1 min of rest between trials, starting with the dominant hand. Two initial practice trials were conducted to familiarize the participant with the test, followed by three attempts with each hand. The result was measured in kilograms of force, calculated as the average of the three attempts for each hand assessed (Alonso, Ribeiro, et al., 2018).

#### 2.5.7 Short physical performance battery (SPPB)

The test comprises three assessments: static standing balance (scored from 0 to 4 points), usual gait speed (also scored from 0 to 4 points), and lower limb muscle strength, which is evaluated by having the individual stand up and sit down in a chair five consecutive times without using their upper limbs (scored from 0 to 4 points). The total score ranges from zero (worst performance) to 12 points (best performance) and is classified as follows: 0 to 3 points indicate incapacity or very poor performance, 4 to 6 points indicate low performance, 7 to 9 points indicate moderate performance, and 10 to 12 points indicate good performance (Nakano, 2007).

#### 2.5.8 Assessment of dynamic balance and mobility

The Timed Up and Go (TUG) test was performed with and without cognitive tasks. The objective of the test is to measure how many seconds it takes for an individual to stand up from a standard chair (approximately 46 cm seat height and 65 cm armrest height), walk three meters, turn 180°, return to the chair, and sit down again. In the TUG with cognitive tasks (TUG+COG), the procedure is similar to the standard TUG, but the participant must perform a dual task by naming animals aloud. In both tests, the time to complete the task is recorded in seconds (Alonso, Brech, et al., 2018).

#### 2.5.9 Senior fitness test (SFT)

The test consists of six physical assessments with specific objectives. The first and second tests evaluate the strength and endurance of the lower and upper limbs, respectively, by measuring the number of repetitions of leg raises and forearm curls. The third and fourth tests assess the flexibility of the lower and upper limbs, respectively, by measuring the ability to reach the floor (in centimeters) and to reach behind the back. The fifth test involves getting up from a chair, agility, and dynamic balance (walking 2.44 meters), and sitting down again, assessing physical mobility and dynamic balance through the time taken. The sixth test evaluates aerobic endurance through a 6‐min walk, measuring the distance covered (Evangelista et al., 2021).

#### 2.5.10 Cardiopulmonary exercise test

The cardiopulmonary exercise test was conducted by an experienced physiologist using a progressive ramp‐style protocol on a treadmill, lasting between 8 and 15 min, and adapted to the individual capacity of each participant. Oxygen consumption (VO_2_), carbon dioxide production (VCO_2_), heart rate via a 13‐lead electrocardiogram, blood pressure, and ventilatory parameters were monitored, with breath‐by‐breath respiratory measurements obtained through a computerized system. The cardiorespiratory optimal point (COP), ventilatory thresholds 1 and 2 (VT1 and VT2), and maximal oxygen consumption (VO_2_max) were determined based on multiple physiological and subjective criteria, including the Borg scale for perceived exertion. This evaluation guided the prescription of aerobic training, ensuring safety and appropriateness to the functional capacities of older adults with T2DM (Bastos et al., 2020).

### 2.6 Allocation, blinding, and loss to follow‐up

Group allocation was performed by a nurse after tests classified participants as neuropathic or nonneuropathic. This classification was kept confidential and stored in a location inaccessible to the blinded evaluators.

Double blind: Both the evaluator who performed the tests and the one who performed the interventions were blinded to the group.

Data entry, data processing, and statistical analysis of all assessment records were conducted by an independent researcher blinded to the group assignments.

Losses to follow‐up were handled using the intention‐to‐treat approach, which is considered the appropriate analysis for superiority clinical trials. In this approach, individuals are analyzed according to their original allocated groups regardless of the treatment actually received, avoiding confounding bias caused by excluding nonadherent patients (per‐protocol analysis).

**FIGURE 1:**
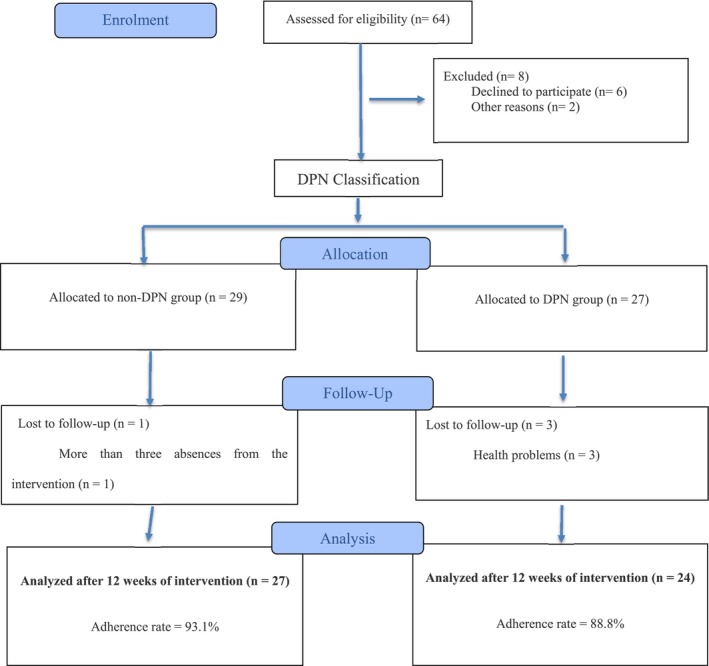
Flowchart of the study stages.

This study recruited 64 participants, of whom eight were excluded: six for not meeting the inclusion criteria and two due to withdrawal. After exclusions, the non‐NDP group had 29 participants and the NDP group had 27. During the intervention, there was one dropout in the non‐DPN group due to a participant exceeding three absences, while the DPN group had three dropouts due to health reasons. At the end of the study, the non‐DPN group remained with 27 participants and the DPN group with 24 (Figure 1).

### 2.7 Intervention

Although gaps still exist in the literature regarding the optimal exercise order specifically for older adults with T2DM, we opted for RT followed by aerobic training, as this sequence was expected to allow better strength performance because participants were not fatigued by prior aerobic activity.

#### 2.7.1 Resistance exercise training

For 12 weeks, training sessions were held twice per week. Six exercises targeting major muscle groups were performed, with 3 sets of 8 to 12 repetitions each. Intensity was monitored using the Rating of Perceived Exertion (RPE) scale ranging from 0 to 10, in accordance with recommendations from the American College of Sports Medicine (American College of Sports Medicine, 2009). If participants were able to perform more than 12 repetitions in two consecutive sessions, the weight load was adjusted. Rest intervals between sets and exercises ranged from 1 to 2 min, under the direct supervision of a trained qualified professional (Lagally & Robertson, 2006). Furthermore, participants were encouraged and instructed to perform 30‐min walking sessions on days not consecutive to the CT.

#### 2.7.2 Interval aerobic training

In this study, the interval aerobic training protocol was performed on a stationary bicycle, starting with 20‐min sessions during the first 2 weeks and progressing to 30 min in the following weeks. Load progression occurred every 2 to 3 weeks, with an approximate increase of 10%. The protocol consisted of interval bouts: 3 min at low intensity (corresponding to the optimal cardiorespiratory point—OCR, approximately 50% of maximum heart rate—HR) followed by 2 min at a ramped intensity corresponding to the second ventilatory threshold (VT2, approximately 70% of HR). Exercise intensity was monitored using a heart rate monitor, and the perceived exertion was assessed using the Borg scale. A rest interval of approximately 5 to 10 min was adopted between the aerobic and RT, adjusted according to the participant's recovery perception, monitored by the Borg scale and/or heart rate.

### 2.8 Statistical analysis

The statistical software Jamovi® was used for the analyses. The Shapiro–Wilk and Levene's tests were applied to verify the normality and homogeneity of variances. Descriptive analysis included means and standard deviation. Student's *t*‐test was used to compare baseline characteristics for quantitative variables. Categorical variables were expressed as absolute and relative frequencies, and the chi‐square test and Fisher's exact test were used to verify associations between categorical variables between groups.

All data were analyzed using generalized linear models (GLM), with participants as random effects, time as a repeated measure, and group‐by‐time interactions as fixed effects. No data imputation was performed, as GLM can handle missing data through maximum likelihood estimation.

Multiple linear regression analyses were conducted to investigate whether neuropathy and other clinical or sociodemographic factors could influence the response in any of the physical and functional tests used in the study. The analyses were performed using a stepwise (backward) method, with an inclusion criterion of *p* < 0.05 and an exclusion criterion of *p* > 0.10. Adjusted *R* ^2^ values were used to determine the quality of the generated models. The following independent variables were used in all regression models: presence of DPN (yes/no), sex, age, time since diagnosis, alcohol consumption, glycated hemoglobin level, and body mass index. The variables insulin use and smoking were excluded from the regression model due to the low number of participants who used insulin or who were smokers. Regression analyses were performed for the change (difference between final and baseline assessments) in the following dependent variables: SFT chair stand test, agility and dynamic balance (2.44 m walk time SFT), 6‐min walk distance (SFT), handgrip strength of the dominant hand, 4‐m walk time (SPPB), TUG without cognitive tasks, and TUG with cognitive tasks. Residual independence was tested using the Durbin‐Watson test, and multicollinearity was assessed using the variance inflation factor (VIF). A significance level of 5% was adopted for all analyses.

## 3 RESULTS

As expected, there was only a difference in the degree of DPN variable between the groups (*p* < 0.001). No statistically significant differences were found between the groups for the other characterization variables (Table 1).

**TABLE 1:** Characterization of the non‐DPN and DPN groups participating in the study.

	Non‐DPN	DPN	*p*‐Value
	M (SD)	M (SD)	
Age (years)	71.63 (4.45)	70.21 (5.09)	0.29
Time since T2DM diagnosis (years)	16.04 (9.97)	20.42 (13.70)	0.19
Degree DPN	1.27 (0.67)	5,64 (2.13)	**< 0**.001*
BMI (kg/m^2^)	26.45 (6.98)	29.44 (4.39)	0.07
Glucose (mg/dL)	120.26 (38.98)	130.33 (34.21)	0.33
Insulin (μU/mL)	12.32 (13.68)	14.8 (8.28)	0.46
HbA1c (%)	6.93 (1.18)	7.04 (1.2)	0.73
Fructosamine (μmol/L)	303.85 (46.68)	300.25 (51.28)	0.79

*Note*: Student's *t*‐test *p < 0.05. Bold values denote statistical bias.

Abbreviations: BMI, body mass index; DPN, diabetic peripheral neuropathy; HbA1c, glycated hemoglobin; M, mean; non‐DPN, without diabetic peripheral neuropathy; SD, standard deviation; T2DM, type 2 diabetes mellitus.

The DPN group used insulin more frequently compared to the non‐DPN group. Regarding the other sociodemographic variables analyzed, no significant differences were identified between the groups (Table 2).

**TABLE 2:** Sociodemographic characteristics of older adults in the non‐DPN and DPN groups.

	Non‐DPN		DPN		*X* ^2^	*p*‐Value
	*n*	%	*n*	%		
Sex						
Men	16	59.3	14	58.3	0.00	0.94
Women	11	40.7	10	41.7		
Physical activity^a^						
Yes	20	74.1	14	58.3	1.42	0.23
No	7	25.9	10	41.7		
Uses insulin?						
Yes	3	11.1	8	33.3	3.71	0.05*
No	24	88.9	16	66.7		
Uses tobacco?						
No	16	59.3	8	33.3	3.89	0.14
Former smoker	10	37.0	13	54.2		
Current smoker	1	3.7	3	12.5		
Consumes alcoholic beverages?						
No	15	55.6	11	45.8	0.48	0.48
Yes	12	44.4	13	54.2		
Continuous use of antidiabetic medications						
Biguanides	14	45.2	17	54.8	1.92	0.16
Sulfonylureas	5	50.0	5	50.0	0.04	0.83
DPP‐IV inhibitors	3	75.0	1	25.0	0.84	0.35
Combination therapy	2	100.0	0	0.0	1.85	0.17
SGLT2 inhibitors	6	54.5	5	45.5	0.01	0.90
Others	3	33.3	6	66.7	1.69	0.19

*Note*: Chi‐square test *p < 0.05. Bold values denote statistical bias.

Abbreviations: %, percentage; DPN, diabetic peripheral neuropathy; *n*, number; non‐DPN, without diabetic neuropathy; *χ* ^2^, chi‐square.

^a^ Physical activity—self‐reported physical activity by participants: the majority (*n* = 26) reported unsupervised walking (2–3 times/week, ~30 min/session). Five participants reported weekly soccer practice (~45 min), and three reported tai chi chuan practice (30 min, twice weekly).

In the assessment of physical performance, the non‐DPN group showed significant improvements in agility and dynamic balance, as measured by the SFT (*p* = 0.01), and in mobility, as measured by the TUG test (p = 0.01). This indicates that participants without DPN responded more effectively to the exercise program in terms of moving quickly and maintaining balance during dynamic tasks. No significant changes were observed in other measures, such as muscle strength or flexibility, suggesting that these aspects were similarly affected in both groups (Table 3).

**TABLE 3:** Comparison of physical performance between the DPN and non‐DPN groups after combined training intervention.

	DPN		Non‐DPN		*p*‐Value
	Pre M (SD)	Post M (SD)	Pre M (SD)	Post M (SD)	
	MD (95% CI)		MD (95% CI)		
Senior fitness test					
30‐s chair stand test (repetitions)	11.21 (2.26)	13.63 (2.48)	12.44 (2.41)	14.74 (3.9)	0.88
	2.42 (1.49 to 3.34)		2.3 (0.90 to 3.69)		
30‐s arm curl test (repetitions)	17.92 (6.1)	23.58 (3.74)	18.07 (4.5)	25.59 (4.6)	0.26
	5.67 (3.19 to 8.14)		7.48 (5.26 to 9.7)		
Sit and reach test (cm)	14.25 (10.84)	10.88 (9.18)	7.02 (8.8)	3.96 (6.55)	0.86
	−3.38 (−6.21 to −0.53)		−3.06 (−5.75 to −0.35)		
Agility and dynamic balance (s)	6.35 (2.08)	6.19 (1.09)	7.16 (1.7)	5.63 (0.86)	0.01*
	0.46 (−0.95 to 1.88)		−1.56 (−2.27 to −0.85)		
Back scratch test (cm)	20.67 (13.27)	16.5 (11.68)	13.57 (7.7)	10.56 (8.94)	0.54
	−4.17 (−7.06 to −1.27)		−3.02 (−5.63 to −0.40)		
6‐min walk test (m)	386.1 (61.0)	445.8 (55.9)	414.5 (64.5)	467.6 (52.5)	0.64
	59.8 (35.6 to 84.0)		53.1 (34.7 to 71.4)		
SPPB					
SPPB score	9.63 (1.52)	11.46 (0.77)	10.41 (1.5)	11.56 (0.69)	0.08
	1.83 (1.17 to 2.49)		1.15 (0.67 to 1.62)		
HGS					
Dominant (Kg/f)	31.27 (8.76)	30.71 (7.49)	30.70 (7.5)	32.67 (8.77)	0.50
	1.04 (−1.68 to 3.77)		1.96 (0.78 to 3.14)		
Nondominant (Kg/f)	29.68 (8.55)	30.58 (6.99)	30.0 (8.07)	31.67 (8.85)	0.72
	2.22 (−0.77 to 5.2)		1.67 (0.18 to 3.15)		
TUG					
TUG (s)	8.55 (1.72)	7.7 (2.56)	8.36 (1.57)	6.4 (0.99)	0.01*
	−0.85 (−1.66 to −0.04)		−1.96 (−2.47 to −1.44)		
TUG + COG (s)	9.98 (2.2)	8.07 (1.01)	10.29 (3.21)	7.53 (1.44)	0.17
	−1.9 (−2.65 to −1.16)		−2.76 (−3.75 to −1.76)		

*Note*: Generalized linear models test *p < 0.05. Bold values denote statistical bias.

Abbreviations: CI, confidence interval; HGS, handgrip strength; M, mean; MD, mean difference; SD, standard deviation; SPPB, short physical performance battery; TUG, timed up and go; TUG+COG, timed up and go with cognitive task.

The linear regression analysis revealed that specific participant characteristics partially explained the variability in physical performance outcomes. For the 30‐s sit‐to‐stand test, BMI and diabetes duration together accounted for 15% of the observed change, indicating that higher BMI and longer diabetes duration were associated with smaller improvements in lower‐body strength. Regarding agility and dynamic balance, the presence of diabetic peripheral neuropathy explained 13% of the variation in SFT performance and 20% in TUG performance, suggesting that neuropathy significantly limits gains in these domains. Finally, for gait speed measured by the 4‐meter walk test, age and BMI explained 12% of the variation, highlighting the influence of older age and higher BMI on walking performance (Table 4).

**TABLE 4:** Final models for the multiple regression analyses.

Dependent variable	Independent variables	Unstandardized coefficient		Standardized coefficient	*t*‐test	*p*	ANOVA		Adjusted *R* ^2^
		*B*	SE	*β*			F (df; residuals)	*p*	
30‐s chair stand test (repetitions)	Constant	8.593	1.962		4.381	<0.001	5.67 (2; 48)	0.05*	0.157
	Time since diagnosis (years)	−0.0679	0.032	−0.281	−2.150	0.037*			
	BMI (kg/m^2^)	−0.179	0.064	−0.368	−2.820	0.007*			
Agility and dynamic balance(s)	Constant	−1.564	0.526		−2.973	0.005	7.28 (1; 43)	0.01*	0.134
	Presence of neuropathy	2.030	0.752	0.380	2.697	0.010*			
4‐m walk test (s)	Constant	1.284	1.605		0.800	0.428	4.85 (2; 48)	0.012*	0.134
	Age (years)	−0.044	0.021	−0.275	−2.085	0.042*			
	BMI (kg/m^2^)	0.036	0.017	0.284	2.154	0.036*			
TUG (s)	Constant	−1.781	0.352		−5.054	<0.001*	5.99 (1; 49)	0.001*	0.209
	Presence of neuropathy	1.107	0.452	0.330	4.448	0.018*			

*Note*: Multiple Regression *p < 0.05. Bold values denote statistical bias.

Abbreviations: df, degrees of freedom; SE, standard error; TUG, timed up and go.

## 4 DISCUSSION

The main findings of the present study indicate that the presence of older adults with DPN is associated with reduced responsiveness to training, particularly in agility, mobility, and dynamic balance performance. This finding suggests DPN in older adults compromises their ability to generate efficient motor responses in tasks requiring rapid postural adjustments, coordinated balance control, and precise movement speed regulation.

DPN primarily affects nerve fibers, impairing the efficient transmission of sensory signals, particularly those related to vibration, movement speed, and rapid changes in joint position (Lindholm et al., 2019). As a result, individuals with DPN may experience difficulties in tests involving mobility, balance, and speed, as their ability to detect and quickly respond to postural changes is compromised (Anandhanarayanan et al., 2000; Holmes & Hastings, 2021). These findings are consistent with the present study, as the tests for mobility, agility, and dynamic balance showed significant improvement in the non‐DPN group. It can therefore be inferred that the relationship between DPN and reduced responsiveness to training may stem from impairments in proprioceptive organs, particularly rapidly adapting receptors such as Pacinian corpuscles, which detect acceleration and dynamic changes, and Ruffini endings, which provide information about joint position and rotational velocity. Both receptor types play a crucial role in dynamic balance, rapid postural adjustments, and agile motor responses, which highlights the need for specific sensorimotor training (Alonso & Vieira, 2007; Maronesi et al., 2016).

These results are also consistent with the findings of Ernandes et al. (2020), which have demonstrated that DPN negatively affects postural and functional balance, impairing motor responsiveness in tasks that require quick adjustments, such as changing direction and maintaining stability when climbing stairs. These deficits stem from reduced plantar sensation and decreased ankle strength and mobility, factors that hinder postural adaptation and the selection of efficient motor strategies (Lindholm et al., 2019). In the present study, we observed that non‐DPN individuals showed improvement in mobility and speed tests after the CT intervention, suggesting that the preservation of sensory and proprioceptive integrity enhances the responsiveness to training. Conversely, postural instability and longer times to complete tasks requiring dynamic gait control in individuals with DPN increase their vulnerability to falls and functional limitations (Callaghan et al., 2012; Ernandes et al., 2020).

The TUG test, as a dynamic functional assessment, requires an efficient reflex response from the lower limb muscles, including the knee flexors and extensors, to ensure stabilization of the center of gravity during movement execution. The action of the quadriceps, responsible for knee extension, and the flexor muscles, which control weight transfer, are crucial for movement efficiency (Evangelista et al., 2024). The improvement observed in the non‐DPN group suggests enhanced postural control and mobility, favoring fall prevention and promoting greater functional independence. These results highlight the importance of strength training for knee flexors and extensors, which are essential for stabilizing the center of gravity during movement (Bohannon, 2006). The present program included strength exercises targeting the quadriceps and hamstrings, as well as trunk exercises, contributing to enhanced neuromuscular coordination, improved postural stabilization, and a reduced risk of falls.

In the regression analysis, for the agility and dynamic balance (SFT) and 3 m (TUG) walking distances, the presence of DPN was identified as a predictor of reduced performance. Our findings are consistent with the study by Metz et al. (2020), who reported that individuals with DPN exhibit greater variability in center of mass (CoM) displacement in both anteroposterior and mediolateral directions. This instability compromises postural control, increases the metabolic cost of walking, and leads individuals with DPN to adopt a more conservative gait pattern as a compensatory strategy, primarily by reducing walking speed, thereby mitigating the impact of sensory and motor deficits (Van Sloten et al., 2011). DPN directly affects sensorimotor adaptability, making it harder to respond to environmental variations during gait. This may reflect alterations in neural control complexity at multiple levels, impairing the integration of sensory and motor feedback necessary for timely postural adjustments and efficient locomotion (Metz et al., 2020; Van Sloten et al., 2011).

In the 4‐meter walk test (SPPB), increasing age and BMI were identified as predictors of poorer performance, which may be attributed to physiological changes associated with aging and excess body weight. Aging leads to a decline in muscle strength and mass, motor coordination, and gait speed (Ko et al., 2011), while a high BMI places greater strain on the joints and is associated with sarcopenic obesity, a common condition among older adults with T2DM (Boonpor et al., 2023). These combined factors impair functional mobility, even in simple, short‐duration walking tasks.

Longer time since diagnosis of T2DM and higher BMI values were predictors of worse performance in the 30‐s sit‐to‐stand test. Longer disease duration is associated with progressive loss of muscle mass and strength, as well as with complications arising from the condition (Sociedade Brasileira de Diabetes, 2023a). This effect may be mediated by the chronic impact of hyperglycemia on muscle tissue and by the worsening of complications such as DPN, which further impairs neuromuscular function and the ability to perform simple functional tasks, such as sitting down and standing up from a chair (Sociedade Brasileira de Diabetes, 2023b).

Higher BMI values, a modifiable factor, were negative predictors of functional progress, a finding consistent with other studies in which individuals with T2DM and overweight or obesity may experience more pronounced progression of DPN symptoms due to excess weight (Nebuloni et al., 2020; Van Sloten et al., 2011). Meanwhile, weight loss and lifestyle modification, especially through an exercise program, have the potential to reduce these symptoms, reflecting the relationship between metabolic factors, glycemic control, and DPN severity (Look AHEAD Research Group, 2017; Singleton et al., 2015).

The absence of significant differences in muscle strength between individuals with and without DPN observed in the present study may be explained by the pervasive systemic effects of T2DM on neuromuscular function. Pathophysiological mechanisms such as diabetes‐related myopathy, insulin resistance, mitochondrial dysfunction, and reduced muscle quality are known to impair muscle performance independently of neuropathy status. In addition, chronic hyperglycemia induces peripheral nerve alterations, including demyelination and motor axon atrophy, which compromise nerve conduction, reduce regenerative capacity, and promote the loss of motor units (Balducci et al., 2006; Nebuloni et al., 2020). These mechanisms likely contribute to the blunted strength‐related adaptations observed in both groups, suggesting that T2DM itself exerts a dominant negative effect on muscle function. Nonetheless, evidence from a recent systematic review and meta‐analysis by Nakashima et al. (2025) involving 23 studies and 2798 participants indicates that individuals with DPN exhibit significantly lower lower‐limb muscle strength compared to those without neuropathy, although with low certainty of evidence. Taken together, our findings align with this body of literature and highlight the clinical importance of progressive resistance training as a key intervention to counteract diabetes‐related neuromuscular impairments and preserve functional capacity, even in the presence of peripheral neuropathy (American College of Sports Medicine, 2009; Holmes & Hastings, 2021; Seyedizadeh et al., 2020).

Moreover, the regression analysis showed that the duration of T2DM diagnosis and BMI accounted for 15% of the variance in performance on the 30‐s chair stand test. It is well established that a longer duration of the disease is associated with a progressive loss of muscle mass and strength in older adults with diabetes (Sociedade Brasileira de Diabetes, 2023a). This decline may be mediated by both the direct effects of chronic hyperglycemia on muscle tissue and the worsening of complications such as DPN, which affects neuromuscular function and makes it more difficult to perform simple functional tasks, such as rising from a chair (Sociedade Brasileira de Diabetes, 2023b).

The findings of the present study suggest that CT may help preserve both motor and sensory functions in individuals with T2DM and DPN. This is consistent with Balducci et al. (2006), who demonstrated that aerobic training, even at low intensity, can slow the progression of DPN, potentially through enhanced neural vascularization, increased nitric oxide bioavailability, and regulation of Na^+^/K^+^‐ATPase activity—processes essential for nerve conduction. Resistance training (RT) complements these effects by increasing muscle strength and lean body mass, thereby counteracting DPN‐associated atrophy, while also improving insulin sensitivity and glycemic control, mitigating the deleterious effects of chronic hyperglycemia on peripheral nerves. Furthermore, RT may promote neuroplasticity and peripheral nerve function through indirect mechanisms such as increased local perfusion and the release of neurotrophic factors. Finally, by enhancing postural stability and lower limb function, it contributes to reducing fall risk in this population (Handsaker et al., 2016).

### 4.1 Strengths and limitations, and perspectives for future

This study has several limitations that should be acknowledged. First, it was not possible to determine whether the chronicity or severity of DPN influenced the training response, as neuropathy severity was not systematically quantified, information regarding disease duration was unavailable, and most older adults were unaware of the progressive nature of the condition. In addition, the higher proportion of insulin use observed in the DPN group may reflect greater metabolic disease severity, representing a potential confounding factor.

Another important consideration is that most participants reported engaging in some form of habitual physical activity, predominantly unsupervised walking. Although no significant differences were observed in baseline activity levels between groups and none of the participants were enrolled in structured exercise programs at baseline, the potential influence of habitual physical activity on neuromuscular function and the progression of DPN cannot be entirely excluded. To mitigate the impact of baseline variability, the statistical analyses focused on within‐group pre–post changes and between‐group comparisons.

A further limitation relates to the frequency of the supervised CT program, which was performed only twice per week. Although this frequency aligns with the ADA recommendations for individuals with type 1 and type 2 diabetes, it may have constrained the magnitude of physiological adaptations that could be achieved with higher weekly training volumes. However, increasing training frequency in older adults with diabetes is often challenging due to comorbidities, functional limitations, and barriers to long‐term adherence.

Future studies should adopt longitudinal designs and incorporate objective measures of neuropathy severity, detailed characterization of diabetes duration and treatment, and comprehensive neuromuscular assessments. Such approaches would allow a more precise understanding of exercise‐induced adaptations across different stages of DPN.

### 4.2 Practical implications

The findings of this study highlight the importance of considering the presence of DPN in the prescription and planning of physical training programs for older adults with T2DM. The improvement observed in the non‐DPN group reinforces the potential of CT to enhance mobility and reduce fall risk in this population. However, the limited response in the DPN group indicates the need for specific therapeutic strategies, such as complementary interventions focused on neuromuscular rehabilitation, progressive strengthening, and sensorimotor training. These findings may help healthcare professionals design more individualized approaches to improve functionality and quality of life in patients with T2DM and DPN. The innovative nature of this research lies in its unprecedented demonstration of how DPN modulates the response to exercise in older adults with T2DM, highlighting gaps and opportunities for optimizing interventions. These findings have the potential to influence future clinical guidelines, promoting more individualized and evidence‐based approaches to improving functionality and quality of life in this population.

## 5 CONCLUSION

The presence of DPN with older adults significantly impairs the responsiveness to CT, particularly in mobility, balance, and agility. Nevertheless, the observed benefits across both groups underscore CT's role as a feasible and effective intervention for older adults with T2DM, regardless of neuropathy status. These findings underscore the need for targeted therapeutic approaches for older adults with DPN, considering specific interventions aimed at mitigating functional limitations and optimizing the benefits of physical training.

## AUTHOR CONTRIBUTIONS

This investigation was made possible by the efforts of a multidisciplinary and international team. Conceptualization, MHSL, ACA; JMDAG Methodology, MHSL, ACA, MVG; Formal analysis, ACA, EYS; Data curation/analysis, GCB and ACA; Writing – original draft preparation, MHSL, ASS, GCB, SRR; Writing – review and editing, ACA, GCB; Supervision, ACA; Funding acquisition, AMSL, GCB, JMDG, ACA. All authors have read and agreed to the published version of the manuscript.

## FUNDING INFORMATION

This study was financed by the Fundação de Amparo à Pesquisa (Foundation for Research Support) process no. 2020/14516‐2. This study was financed in part by the Coordenação de Aperfeiçoamento de Pessoal de Nível Superior—Brasil (CAPES)—Finance Code 001.

## CONFLICT OF INTEREST STATEMENT

The authors declare that they have no competing interests.

## Data Availability

The data will be made available upon reasonable request to the corresponding author.
